# Mycobacteriophage Rita: a cluster F1 phage discovered in North Easton, Massachusetts

**DOI:** 10.1128/MRA.00510-23

**Published:** 2023-08-28

**Authors:** Anna M. Fakhri, Marcie H. Warner, Joseph A. DeGiorgis, Kathleen Cornely

**Affiliations:** 1 Department of Chemistry and Biochemistry, Providence College, Providence, Rhode Island, USA; 2 Department of Natural Sciences, University of Pittsburgh, Pittsburgh, Pennsylvania, USA; 3 Department of Biology, Providence College, Providence, Rhode Island, USA; 4 Whitman Center, Marine Biological Laboratory, Woods Hole, Massachusetts, USA; Loyola University Chicago, Chicago, Illinois, USA

**Keywords:** bacteriophages, genome analysis

## Abstract

Mycobacteriophage Rita infects *Mycobacterium smegmatis* mc^2^155 and was isolated from a soil sample collected in North Easton, Massachusetts. Assigned to cluster F1 based on sequence similarity to other phages in the same cluster, Rita has a 58,771 bp genome and encodes 104 genes. Rita is 98% similar to phage Bipolar.

## ANNOUNCEMENT

Mycobacteriophages have recently been utilized therapeutically against pathogenic and antibiotic-resistant mycobacteria (1, 2). Therefore, mycobacteriophage discovery is a crucial tool in the advancement of phage therapy (3, 4).

**Fig 1:**
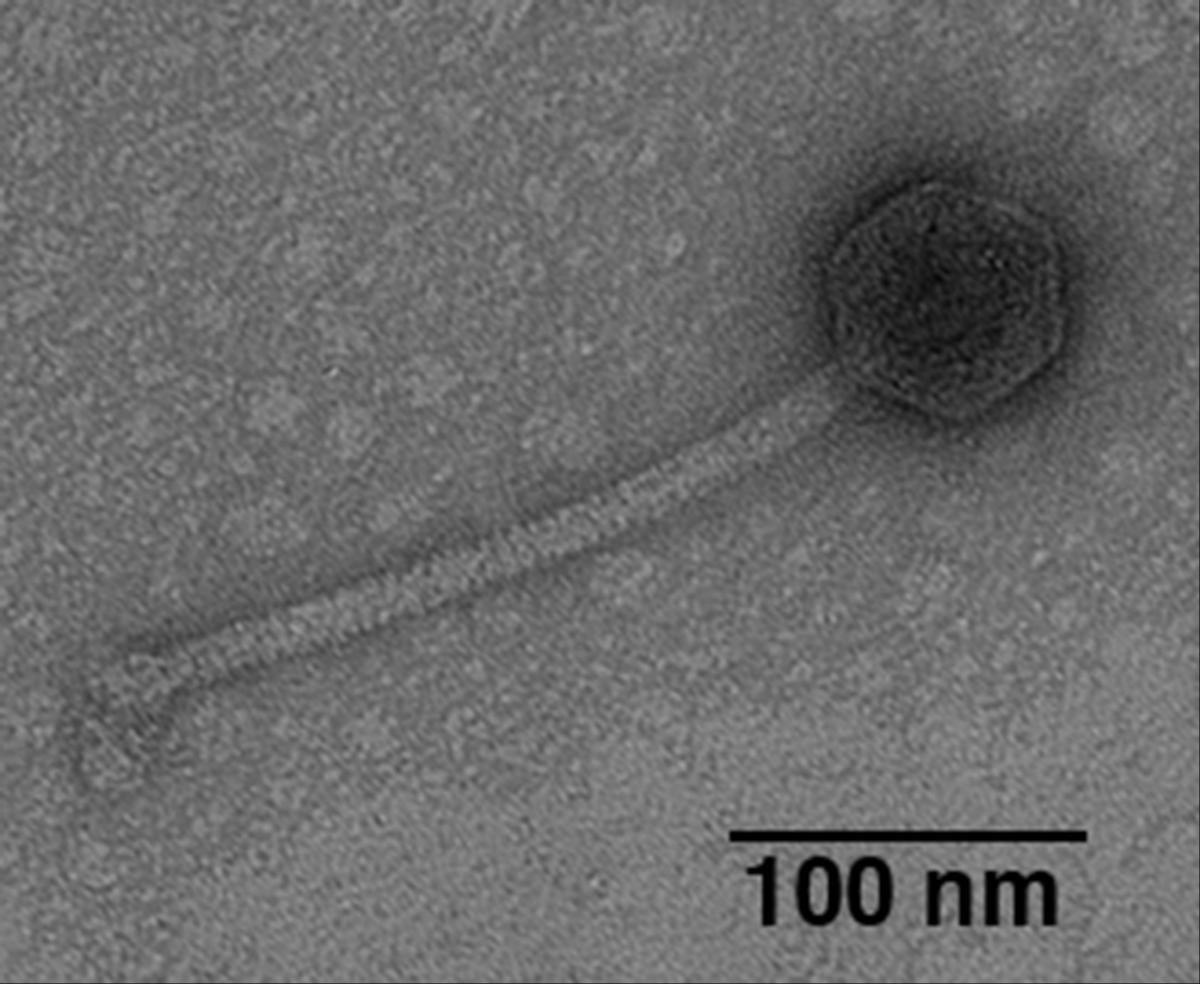
Image of negative-stained (1% uranyl acetate) Rita taken by a JEOL 200 CX transmission electron microscope, identifying Rita as a siphovirus, with an icosahedral capsid 72–75 nm in diameter and a tail length of 190–250 nm (*n* = 6 particles).

Rita was isolated from damp, peaty surface soil underneath an apple tree (Table 1), utilizing standard methods (5). The soil sample was washed in 7H9 liquid medium and filtered (0.22 µm pore size), and the filtrate was inoculated with *Mycobacterium smegmatis* mc^2^155. After overnight incubation with shaking at 37°C, the filtrate was plated in top agar with *M. smegmatis* to yield plaques of phage Rita. Rita, which was purified through five rounds of plating, forms clear plaques <1 mm in diameter. Rita exhibits siphovirus morphology as determined using negative-stain transmission electron microscopy (Fig. 1; Table 1). Phage DNA was isolated from a high-titer lysate by phenol:chloroform:isoamyl alcohol extraction (6) and sequenced at the Pittsburgh Bacteriophage Institute (Table 1). Reads were verified for accuracy using Consed v29.0 (7) and assembled using Newbler v2.9 (8). Rita’s genome is 58,771 bp in length with a 3′ single-stranded overhang and a GC content of 61.6%. Rita was assigned to subcluster F1 based on gene content similarity of >35% to F1 phages Bipolar (KM597530), Kenuha5 (MN369739), and SuperGrey (KX808131) in the Actinobacteriophage database (phagesDB) (9 - 12).

**TABLE 1:** Sequencing, genome, and phage characteristics

Parameter	Phage data
Soil sample characteristics	
Collection date	28 May 2021
Collection location coordinates	42.051462N, 71.134382W
Phage particle characteristics	
Capsid size (nm)	72–75 (*n* = 6)
Tail length (nm)	190–250 (*n* = 6)
Sequencing	
Sequencing instrument	Illumina MiSeq v3 reagents
Library prep kit	NEB Ultra II Library Kit
No. of reads	1,122,251
Length of reads (bp)	150 base single end reads
Shotgun coverage (×)	2,788
Phage genome characteristics	
Genome length (bp)	58,771
3’ single-stranded overhang	10 bases (5′-CGGAAGGCGC-3′)
GC content (%)	61.6%
attP site (bp)	31,855–31,880

The genome sequence was annotated using DNA Master v5.22.2 (13), Glimmer v3.02 (14), GeneMark v2.5 (15), and Starterator v1.1 (16) to determine start and end sites. tRNAscan-SE (17) and Aragorn (18) did not identify any tRNAs in the genome sequence. Protein-coding genes were assigned functions using data from the NCBI Conserved Domain Database (19), HHpred (20), TMHMM v2.0 (21), TOPCONS (22), and BLASTp v2.2.26 (23), as well as genome comparison to other phages in the F1 subcluster using Phamerator (24). Of the 104 protein-coding genes identified, 41 were assigned a function.

A variety of genes encoding phage structure and assembly functions are apparent in Rita which are found on the left half of the genome. These are followed by a lysis cassette consisting of lysin A, lysin B, and holin proteins. Genes encoding an immunity repressor (*46*) and tyrosine integrase (*44*) were identified, along with a phage attachment site (attP) at 31,855–31,880, indicating that Rita can potentially adopt a temperate lifestyle. The genome contains two Whib family transcription factors (*58, 60*), two helix-turn-helix DNA-binding domains (*42, 61*), and a DnaQ-like DNA polymerase III subunit (*36*).

Notably, Rita contains a histidine nucleotide triad-binding protein (Hint, *67*) identified in only three other F cluster phages, though Hint proteins have been identified in other mycobacteriophage clusters as well as in Gordonia phages. Hint proteins are members of a superfamily consisting of hydrolases and nucleotidyl transferases (25). The histidine triad motif contains three conserved histidines as catalytic residues. Hint proteins in humans activate nucleoside antiviral and anticancer prodrugs (26), but the function of Hint proteins in phages is unknown. The ubiquity of Hint homologs suggests an essential cellular function.

## Data Availability

The genome sequence accession number is OP068340, and the SRA accession number is SRX15605406.
